# Molecular markers in triple-negative breast cancer: A retrospective correlation study

**DOI:** 10.6026/973206300212245

**Published:** 2025-07-31

**Authors:** Sudarshan Gupta, Purti Agrawal Saini, Vikas Pandey, Mitesh Shah

**Affiliations:** 1 Department of Pathology, VKS Government Medical College, Neemuch, Madhya Pradesh, India; 2 Department of Pathology, Nandkumar Singh Chouhan Government Medical College, Khandwa, Madhya Pradesh, India; 3 Department of Pathology, Government Medical College, Santa, Madhya Pradesh, India; 4 Department of Pathology, Bundelkhand Medical College, Sagar, Madhya Pradesh, India

**Keywords:** Triple-negative breast cancer, Ki-67, p53, EGFR, CK5/6, basal-like phenotype, molecular markers

## Abstract

The expression and clinical correlation of molecular markers in 128 patients diagnosed with triple-negative breast cancer (TNBC). Immunohistochemistry was used to assess markers including Ki-67, p53, EGFR and CK5/6. High Ki-67 and p53 positivity were significantly associated with higher tumor grade and lymph node involvement. EGFR and CK5/6 expression correlated with basal-like features and poor differentiation. Data suggest that molecular profiling in TNBC may guide prognosis and targeted therapies.

## Background:

Due to the fact that the treatment of breast cancer depends significantly on the molecular markers present in the cancer, including estrogen receptor (+), progesterone receptor (+) or erbB2 receptor (+), further investigation targeting triple-negative breast cancer (TNBC) subtypes may assist in elucidating the mechanisms of recurrence of TNBC and enable the identification of novel therapeutic strategies for patients with TNBC [1]. Triple-negative breast cancer (TNBC) is a biologically aggressive subtype of breast carcinoma defined by the absence of estrogen receptor (ER), progesterone receptor (PR) and human epidermal growth factor receptor 2 (HER2) expressions [2]. It accounts for approximately 15-20% of all breast cancers and is associated with poor prognosis, early metastasis and limited treatment options due to the lack of targeted hormonal or HER2-directed therapies. Given this therapeutic void, identifying molecular markers that define the biological behavior of TNBC is crucial [3]. There are many biomarkers in TNBC being used in clinical practice. Biomarkers may be useful as prognostic or predictive indicators as well as suggest possible targets for novel therapies. Many targeted agents are being studied for treatment of TNBC [4]. Markers such as Ki-67 (a proliferation index), p53 (a tumor suppressor gene), EGFR (epidermal growth factor receptor) and CK5/6 (a basal cytokeratin) have been investigated for their potential prognostic and therapeutic significance [5]. These markers can help distinguish basal-like phenotypes and guide the development of molecular-targeted strategies in TNBC [6]. Therefore, it is of interest to describe the prevalence of selected molecular markers in TNBC and explore their association with clinicopathological features to support personalized management strategies.

## Materials and Methods:

This retrospective study was conducted on 128 histologically confirmed cases of triple-negative breast cancer (TNBC) diagnosed between January 2018 and December 2022 at a tertiary care oncology center. Patient data including age, tumor size, histological grade, lymph node status and recurrence were collected from medical records and pathology archives. Only patients with complete immuno-histochemical (IHC) data and follow-up records were included. All tissue specimens were formalin-fixed and paraffin-embedded and IHC staining was performed using standardized protocols. The molecular markers evaluated included Ki-67, p53, epidermal growth factor receptor (EGFR) and cytokeratin 5/6 (CK5/6). Ki-67 was considered high when ≥20% nuclear staining was observed. P53 positivity was defined as ≥10% nuclear staining. EGFR and CK5/6 were scored positive when ≥10% membranous or cytoplasmic staining was seen in tumor cells. Statistical analysis was carried out using SPSS version 26. Descriptive statistics were used to summarize clinical and pathological features. Associations between molecular marker expression and clinicopathological parameters were analyzed using chi-square tests and Fisher's exact test where appropriate. Multivariate logistic regression was performed to identify independent predictors of recurrence and lymph node involvement. A p-value <0.05 was considered statistically significant.

## Results:

A total of 128 TNBC patients were included, with a mean age of 49.6 ± 10.2 years. Most tumors were high-grade (Grade III: 68.8%) and had a mean size of 3.4 ± 1.1 cm. Lymph node metastasis was observed in 62 cases (48.4%) and 1-year recurrence was documented in 29 patients (22.7%). Expression of Ki-67 (≥20%), p53, EGFR and CK5/6 was noted in 74.2%, 61.7%, 39.8% and 36.7% of cases, respectively. The results below illustrate the distribution and correlation of molecular markers with clinicopathological parameters.

Table 1 (see PDF) presents the age distribution of TNBC patients, showing that the majority were in the 41-60 year age range. There was no significant association between age and the expression of molecular markers, suggesting that age did not influence marker prevalence in this cohort. Table 2 (see PDF) details the distribution of tumor grades and their correlation with Ki-67 and p53 expression. Grade III tumors were most common and showed significantly higher positivity for both Ki-67 and p53, indicating their association with more aggressive histological behavior. Table 3 (see PDF) correlates tumor size with Ki-67 expression. Tumors larger than 2 cm showed a significantly higher frequency of Ki-67 positivity, suggesting that proliferative index increases with tumor size, reflecting more aggressive growth kinetics. Table 4 (see PDF) examines lymph node involvement in relation to molecular markers. Both p53 and EGFR expression were significantly associated with nodal metastasis, indicating their potential prognostic relevance for tumor dissemination. Table 5 (see PDF) compares marker expression in patients with and without recurrence. A significantly greater proportion of recurrent cases expressed high Ki-67 and CK5/6, implicating these markers in early relapse risk. Table 6 (see PDF) explores the relationship between basal-like histological features and EGFR/CK5/6 expression. Tumors with basal morphology showed a significantly higher expression of these two markers, reinforcing their role in characterizing basal-like TNBC phenotypes. Table 7 (see PDF) describes the co-expression patterns of Ki-67 and p53. Over half of the cases were positive for both markers, suggesting a subset of tumors with high proliferative and potentially unstable genomic profiles that may predict aggressive clinical behavior. Table 8 (see PDF) provides multivariate logistic regression analysis identifying independent predictors of lymph node involvement. Ki-67 ≥20%, p53 positivity and EGFR expression were all significant predictors, indicating their collective utility in identifying patients at higher risk for metastasis. Table 9 (see PDF) further illustrates the correlation between tumor grade and mean Ki-67 index. A clear trend was observed, with Ki-67 increasing progressively from Grade I to Grade III, affirming its value as a marker of tumor proliferation and dedifferentiation. Table 10 (see PDF) associates EGFR expression with histological features such as tumor necrosis and high mitotic index. EGFR-positive tumors more frequently exhibited these aggressive histopathological characteristics, supporting EGFR's link to high-grade tumor biology. Table 10 (see PDF) shows EGFR-positive tumors more frequently exhibited necrosis and high mitotic index.

## Discussion:

This retrospective study sheds light on the expression patterns and clinical significance of key molecular markers in triple-negative breast cancer (TNBC), a challenging subtype known for its aggressive behavior and limited therapeutic options [6]. The findings reinforce the notion that TNBC is not a homogeneous entity and molecular heterogeneity can guide risk stratification and potential targeted strategies. Ki-67, a well-established proliferation marker, was positive in over 74% of cases and showed significant association with larger tumor size, higher grade and recurrence [7]. This emphasizes the role of Ki-67 in predicting tumor aggressiveness and poor outcomes [8]. Similarly, p53 overexpression, observed in 61.7% of cases, was significantly linked to lymph node involvement and co-expressed with Ki-67 in more than half the cohort, highlighting a proliferative and genomically unstable phenotype that may be more prone to early metastasis [9, 10]. EGFR and CK5/6, both associated with basal-like differentiation, were found in 39.8% and 36.7% of patients, respectively. Their expression was strongly correlated with histological features such as necrosis and high mitotic index, suggesting their role in tumor proliferation and necrotic progression [11]. Notably, EGFR expression independently predicted lymph node metastasis and was significantly more common in tumors with basal morphology, supporting its utility as both a prognostic and potentially therapeutic biomarker [12, 13]. Multivariate analysis further validated Ki-67 ≥20%, p53 positivity and EGFR expression as independent predictors of lymph node involvement-markers that may help identify high-risk patients within the TNBC spectrum [14, 15]. The study reinforces that integrating molecular profiling into routine histopathological evaluation of TNBC can enhance prognostic accuracy and inform individualized treatment plans [16, 17]. Limitations include its retrospective design and lack of genomic sequencing to further classify TNBC subtypes. Nevertheless, this study supports the clinical utility of immuno histochemical markers in prognostication and underscores the need for prospective trials evaluating marker-driven therapy in TNBC [18].

## Conclusion:

This study highlights the significant association of Ki-67, p53, EGFR and CK5/6 expression with poor prognostic features in triple-negative breast cancer. Ki-67 and p53 were strongly linked to high-grade tumors and lymph node involvement, while EGFR and CK5/6 marked basal-like differentiation and aggressive histology. Incorporating these molecular markers into routine assessment can improve risk stratification and may guide future targeted therapies in TNBC.
